# Methodological Framework: Discovering Foods or Nutrients with a Causative Influence on Body Weight in Free-Living Settings

**DOI:** 10.3389/fnut.2014.00017

**Published:** 2014-10-09

**Authors:** Emily J. Dhurandhar

**Affiliations:** ^1^Department of Health Behavior, Office of Energetics, Nutrition Obesity Research Center, University of Alabama at Birmingham, Birmingham, AL, USA

**Keywords:** obesity, energy balance, causation, policy, food

## Abstract

A significant increase in food availability has been implicated in the worldwide obesity epidemic, and there is significant interest in understanding if specific foods may contribute to weight gain or aid in weight loss. Many foods have been studied in controlled laboratory studies or short-term studies, and these findings are often the basis for food-based public health recommendations to reduce or prevent obesity. Unfortunately, often these findings are not applicable to free-living settings where many interacting factors influence energy balance and body weight. Therefore, a proposed set of evidence criteria for making a food-based public health recommendation to reduce or prevent obesity is outlined and discussed herein, to serve as a basis for future committee or panel discussion and formalization. Using these criteria as a basis for making strong evidence-based recommendations will improve the state of science and policy in this area.

## Introduction

Obesity is the largest public health concern in developed countries, and there is a need to understand if specific foods, food attributes, nutrients, or eating patterns have a causative influence on body weight. This knowledge is needed to inform evidence-based public health policies and guidelines to reduce obesity. The general confusion around the issue of what to eat, and what not to eat, to prevent weight gain or promote weight loss, is reflective of a lack of thorough evidence-based criteria for a food or nutrient being implicated in weight change.

For example, in the 1990s and early 2000s, much of the public health nutrition messaging and regulations for reducing obesity focused on fat (1), whereas more recently, the focus has shifted to refined sugars as culprit in the obesity epidemic (2). This recommendation was based on mostly epidemiological evidence, and was ineffective. Another example of a still widely espoused and frequently implemented public policy is the promotion of fruit and vegetable consumption to reduce population levels of obesity. Fruit and vegetable recommendations are also based primarily on epidemiological evidence, and randomized controlled trials (RCTs) increasing fruit and vegetable consumption have very little effect on weight loss (3). These are examples of policies implemented prior to subjecting them to strict evidence-based criteria. We are not aware of a food-specific policy about weight that has been subjected to rigorous evidence criteria and been successful.

These inconsistencies are some of many in the field of nutrition, and may reflect methodological challenges to establishing causation in nutrition research. Herein, criteria for determining a causative role of a food or nutrient in weight change are outlined. Current challenges to meeting those criteria are discussed, and future research directions in this area are highlighted.

## Food, Energy Balance, and Weight Change: The Importance of Context

The first law of thermodynamics dictates that in order for a food to have a causative influence on body weight, it must have a net effect on energy intake, energy expenditure, or both. Because energy cannot be created or destroyed, in order for an increase in body energy stores to occur, energy intake must exceed energy expenditure. Conversely, for a reduction in energy stores to occur, energy intake must be less than energy expenditure. This is a fundamental law; however, there are many interacting factors that influence energy intake and energy expenditure in free-living humans. Environmental (4), social (5), and underlying biologic factors (6) all influence energy balance and body weight.

Studying the causative influence of foods on body weight in a free-living setting, or in the “real-world,” where individuals are exposed to the full myriad of factors that can influence energy balance, is therefore particularly crucial. Public health nutrition policies are meant to guide the general public in weight management in this setting. For a food or nutrient to cause weight change in either direction in a free-living setting, its effect must be sufficient to have an influence even in the presence of other environmental, social, and underlying biologic factors that also influence energy balance. If the factor of interest is not sufficient to cause weight change in this setting, it is unlikely that any public policy or clinical guideline with related recommendations will be effective in reducing the prevalence of obesity.

Food-related public health guidelines for weight loss, or for maintaining a healthy weight, are often justified by extrapolation of results from controlled laboratory studies of effects of foods on energy intake over single meals or at most, a few days to weight change in real-world settings. For example, suppose that it is found that consuming a sugar-sweetened beverage results in 50 kcal more to be consumed relative to a meal without a sugar-sweetened beverage. Based on this finding, it is assumed that sugar-sweetened beverages will produce weight gain, and the weight gain that may result is calculated. This extrapolation is usually accomplished one of two ways: either the “3500 kcal rule” is used (7) or other mathematical models for prediction of weight change given a certain perturbation in energy balance for a certain period of time are used (8, 9). However, for several reasons neither of these techniques is appropriate for extrapolating a short-term change in energy intake to long-term changes in body weight in free-living individuals.

The 3500 kcal rule comes from the fact that 1 lb of fat contains approximately 3,500 kcal of energy, so a net 3,500 kcal change in energy balance should result in 1 lb of weight change (7). Using this rule to predict or quantify weight change is an oversimplification of energy balance, because it does not account for the fact that energy balance is a dynamic and adaptable system (10, 11). As a result, this rule significantly overestimates weight change (10, 11), and is not appropriate to justify or quantify the impact of a food or nutrient related public health policies to prevent or reduce obesity.

Available mathematical models that are sometimes used for this purpose account for metabolic adaptations that occur with changes in energy balance and body weight (8, 9), but are also likely to overestimate weight change in free-living settings because they do not account for the behavioral adaptations that are likely to occur for perturbations in energy balance in a free-living setting. They were developed to estimate weight change in a highly controlled clinical setting where the perturbation in energy balance occurs in isolation, and all other aspects of energy balance are held constant. For example, these models would be appropriate to predict weight change resulting from adding a daily sugar-sweetened beverage to the diet if food intake from other sources and energy expenditure were held constant. In free-living settings, it is not likely that perturbations in energy balance occur in isolation. In this setting, adding a sugar-sweetened beverage to the daily diet may increase spontaneous energy expenditure, or may result in decreased food intake from other sources. Thus, available mathematical models are also not appropriate for quantifying the potential impact of food-related public health policies to prevent or reduce obesity.

To create evidence-based food-related public health policies and guidelines to reduce or prevent obesity, it is crucial that we subject the food in question to rigorous testing and verify that it meets evidence criteria that are appropriate for evaluating the contribution of the food to weight control in free-living settings. If this is not done, the risk of wasting public health resources, sending mixed messages about what foods will and will not help control body weight, and reducing trust of the general public is high. The criteria outlined below can be used for this purpose.

## Criteria

Several types of evidence can suggest that a food may influence energy balance and weight, and can justify further study of the causative contribution of that food to body weight change. In the following criteria, “food” will be referred to very generally, however, these criteria may be applied to a food, specific nutrient, or food-related factor (e.g., food attributes such as volume or form, timing of food consumption, etc.). If a food meets the following preliminary criteria consistently in several studies by independent groups, it can be considered for further testing:

1. Consumption of the food is associated with body weight or body fatness in epidemiological observational studies.
2. Consumption of the food during a single *ad libitum* meal, from initiation to satiation, changes energy intake from that meal relative to when that food is not offered with the same *ad libitum* meal.
3. Consumption of the food on a given day (or over several days) changes 24-h *ad libitum* energy intake relative to a day (or several days) when that food is not consumed.
4. One or more components of energy expenditure is/are different on days when the food is consumed relative to days it is not consumed.

Obesity public health policies are often made based on one or more of the preliminary criteria 1–4 being met from one or more studies. However, even if all preliminary criteria are met, this is insufficient to conclude a food plays a causative role in determining body weight in a free-living setting. Observational studies cannot determine causation, making evidence for criteria 1 often inconsistent, and insufficient to determine causation. If criteria 2–4 are met through carefully controlled trials, this is also an insufficient basis for an evidence-based policy for many reasons.

Although the ability of a food to have a causative influence on energy intake in a single meal, or either energy intake or expenditure for one to several days is promising and a logical indicator of an effect on weight, these findings do not preclude the possibility that this effect will not persist to an extent necessary to influence body weight over the long-term. Because there are learning effects that occur in response to perturbations in energy balance (12), adaptations may occur to negate any significant weight change. In addition, often a study will demonstrate a change in energy intake *or* energy expenditure following incorporation of a food into the diet, but this only provides information about one variable in the energy balance equation. Because energy balance is a dynamic and adaptable system, it is important to demonstrate that a change in energy intake, for example, is not accompanied by an equivalent and opposite change in energy expenditure, which could actually indicate a net “0” change in overall energy balance. Finally, studies such as those described in criteria 2 are often conducted in a highly controlled laboratory setting, where subjects are not permitted to behave naturally and in a way that would allow them to compensate as they would in a free-living setting, making any extension of these findings to this setting inaccurate.

Methodological challenges in accurately measuring food intake and energy expenditure in free-living settings also preclude consistent observations from studies such as those described in criteria 1–4. Most studies attempting to capture shifts in energy balance over several days after incorporation of a food into the diet measure food intake or energy expenditure through self-report measures. It has long been understood (13) and recently highlighted that self-report dietary recalls are extremely error-prone and an “insufficient basis for scientific conclusions” (14). Energy expenditure is also often measured through inaccurate self-report methods (13). These inadequate methods for finding short-term changes in energy balance as a result of incorporating a food into the diet make it challenging to find consistent evidence that foods influence these outcomes.

However, a RCT conducted meeting specific criteria can overcome all of these methodological downfalls. Therefore, an additional key criterion needs to be met through several replications by independent groups to make an evidence-based public health policy regarding food and body weight:

5. An RCT comparing consumption of at least two objectively verified levels of the food as part of an *ad libitum* diet in a free-living setting, for at least 6 months, finds that a change in consumption of the food causes weight change.

Such an RCT is designed to make a strong conclusion: that a change in consumption of the food in question results in statistically significant weight change. It may be worthy to test the food in several groups in which the food may have different effects, such as children vs. adults vs. elderly. To ensure fidelity of the intervention, it is crucial that adherence to the intervention be objectively measured, and if possible, the intervention should be verified through direct observation. Conducting studies that objectively verify consumption without direct observation is currently not possible, and technologies are in development (15, 16) and are urgently needed to do these types of studies in natural settings and to advance the field. A 6-month duration will account for seasonal effects on weight and long-term energy balance adaptations. Having participants consume the food, but otherwise maintain their typical diet and exercise habits, allows for the full range of energetic compensation and gives this type of study high-external validity. If weight change is the primary outcome, it negates any need for error-prone food intake and energy expenditure measures as intermediate indicator outcomes. This fifth and final criterion for making an evidence-based policy about food and weight is robust and appropriate for drawing conclusions about causation in free-living populations.

It is noteworthy that several indicators often used as evidence that a food may influence body weight are not included on the criteria list. For example, the influences of a food on subjective feelings of satiety, fullness, or hunger are not included. It is not clear if increased feelings of satiety or hunger always translate to measureable energy intake outcomes. For example, peanut butter produces less satiety than whole peanuts, but peanut butter produces more adequate energetic compensation (151%) than whole peanuts (104%) (17). In addition, the ability of a food to influence some physiological process of metabolism, for example, if a component of the food increases lipolysis, is not on the criteria list. Although such measures can be informative in some cases for understanding mechanisms and other outcomes, since they do not consistently translate to substantial and measureable changes in energy intake or energy expenditure, they are not included.

## Conclusion and Future Directions

The criteria outlined herein may serve as a basis for a formal panel or workshop discussion to formalize evidence criteria to determine the causative influence of foods on body weight. In order to understand the influence of foods on body weight in free-living populations, because of the nature of energy balance and what impacts it, it is imperative to study the influence of foods in this context. Improvements in technology to objectively and accurately measure food intake and energy expenditure in free-living settings would significantly advance research in this area. Until we understand what foods have an influence in this context, it will be difficult to implement effective food-based policies for prevention and treatment of obesity in the general public and population the policies are intended for.

## Conflict of Interest Statement

The author declares that the research was conducted in the absence of any commercial or financial relationships that could be construed as a potential conflict of interest.
